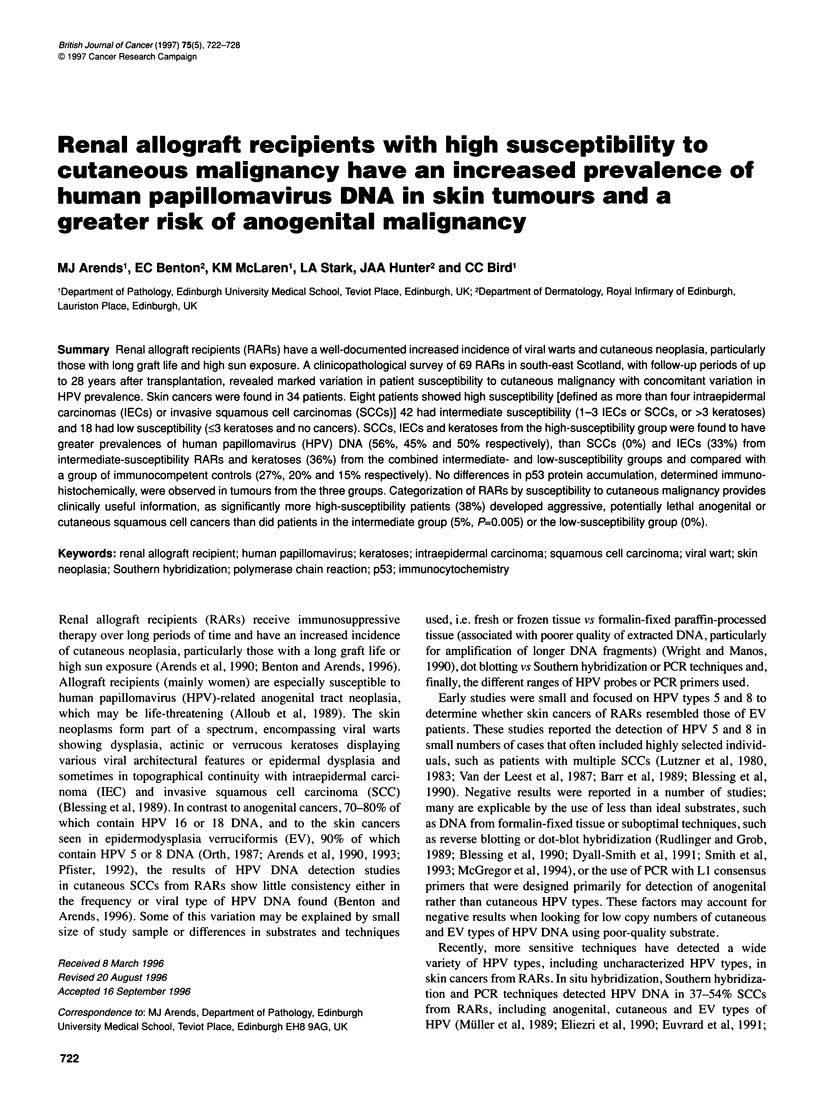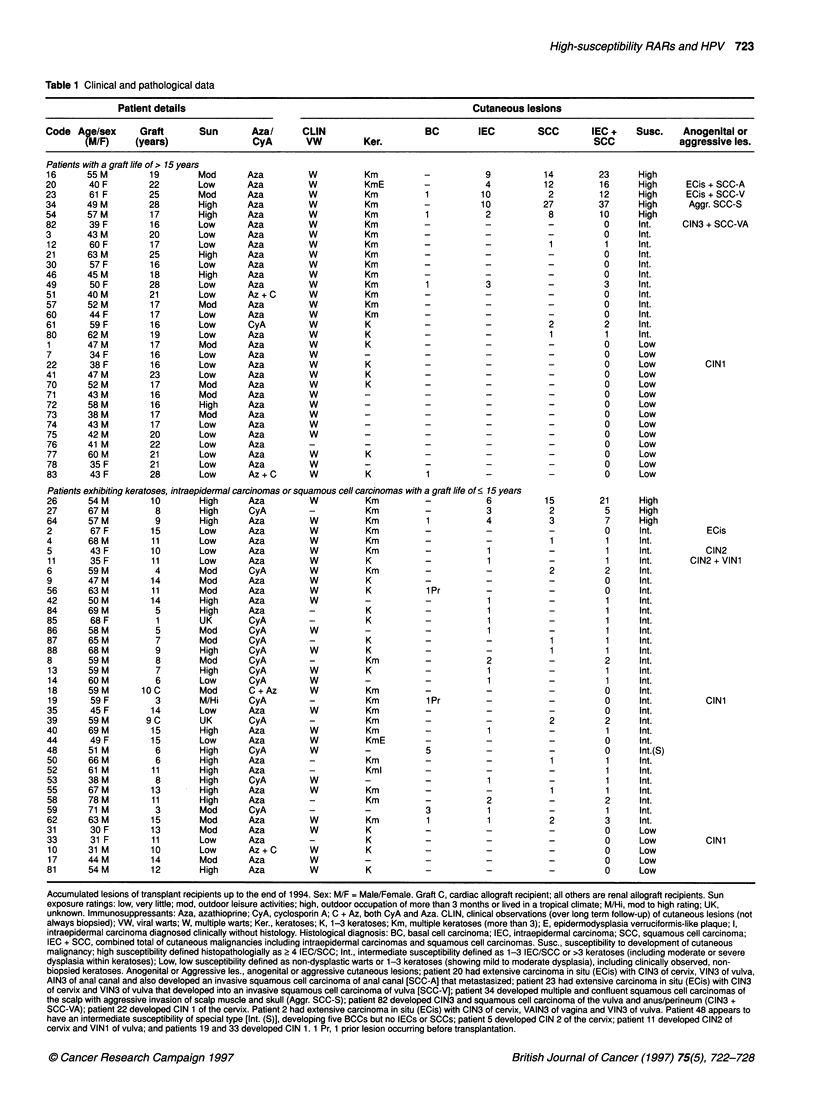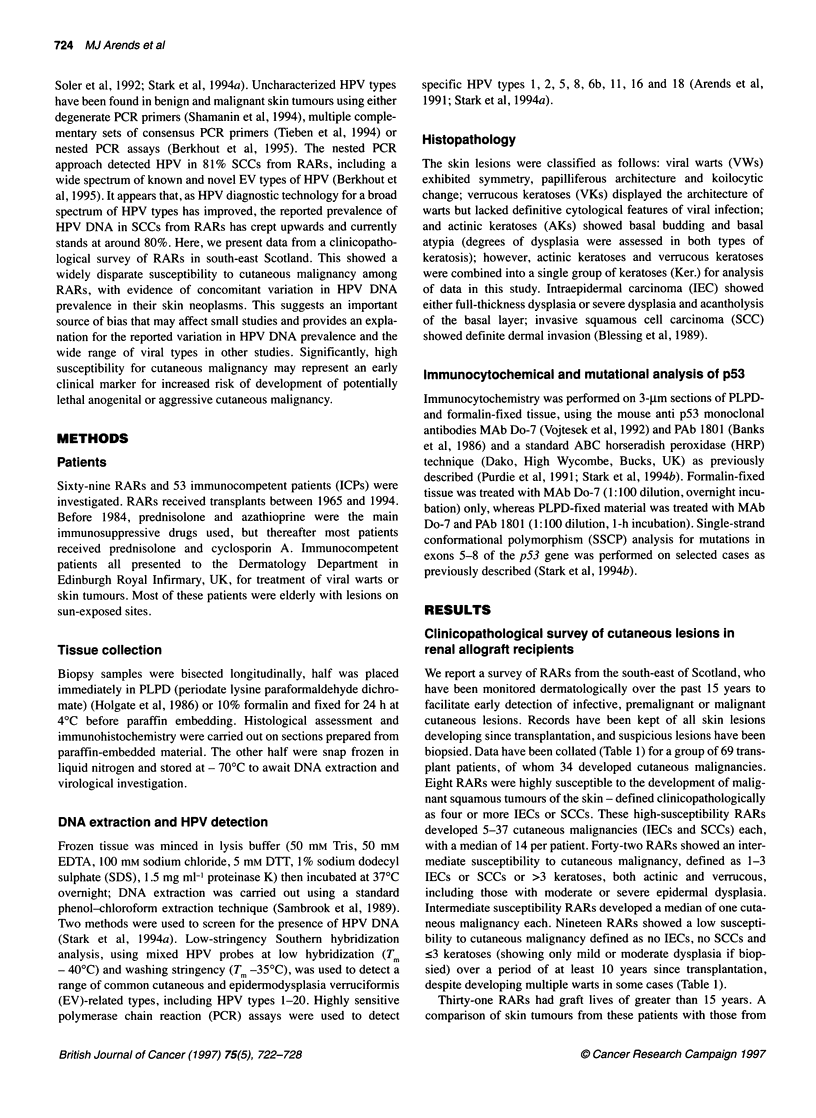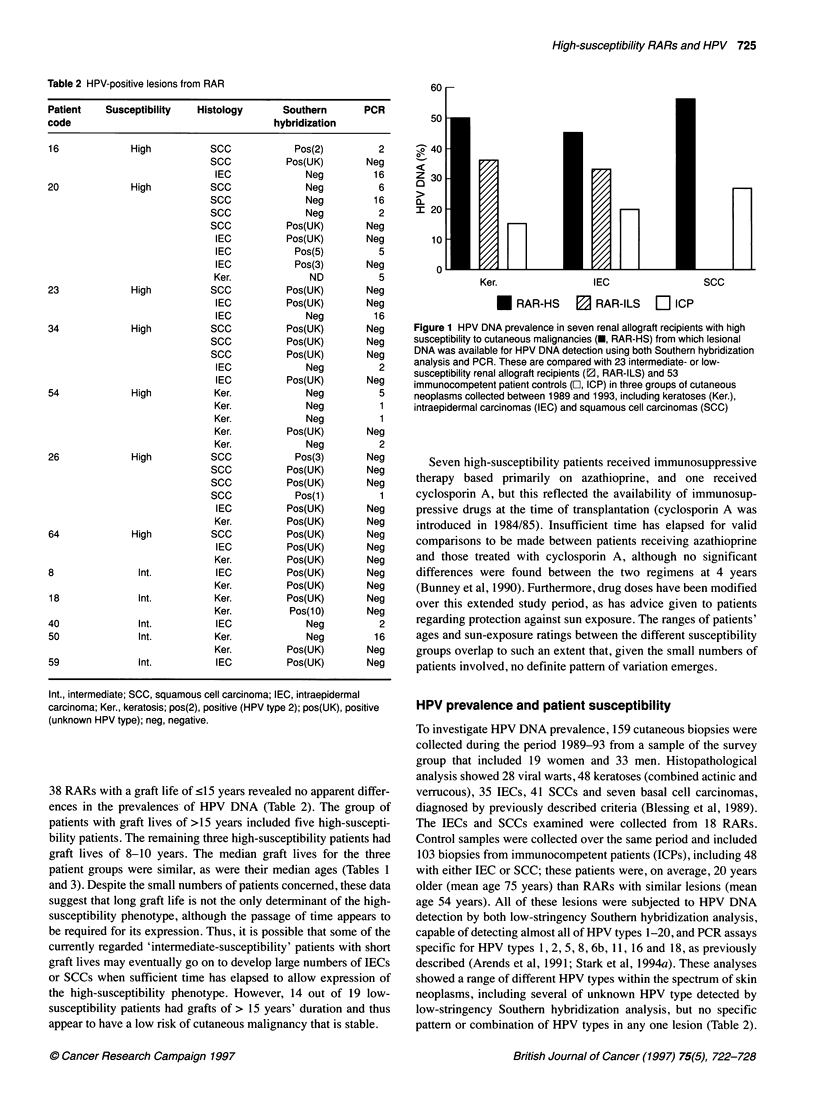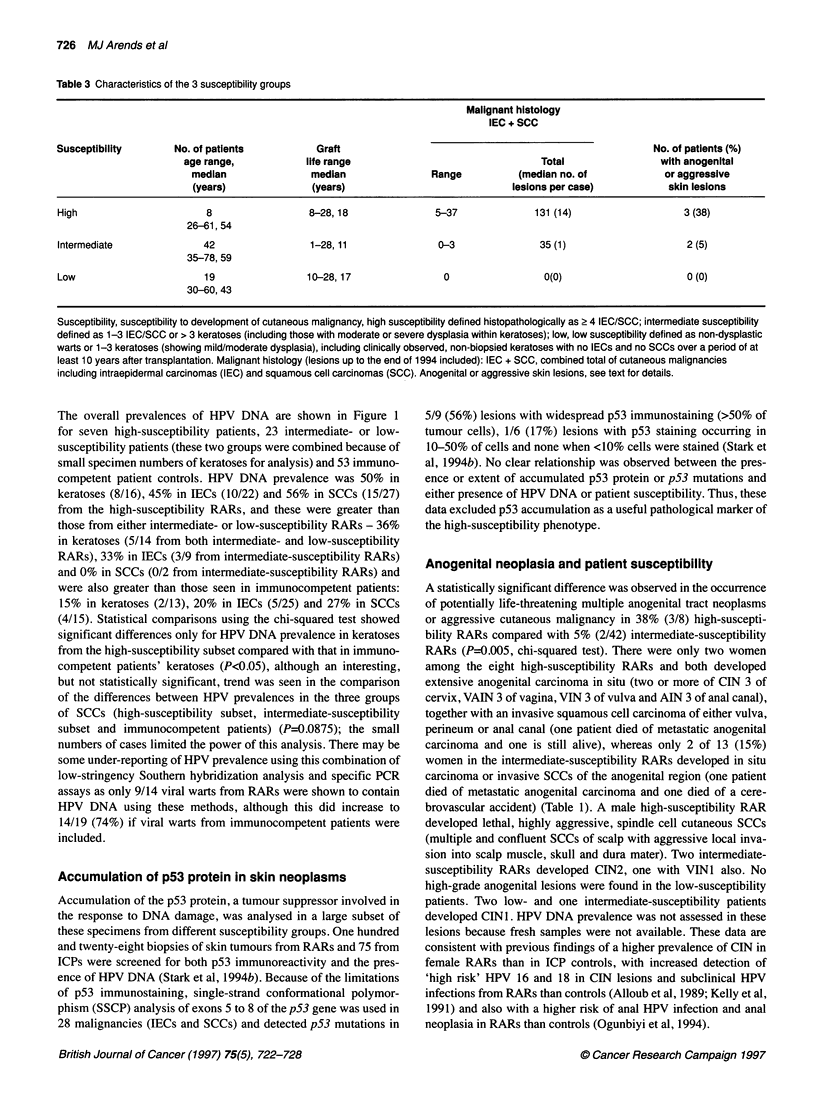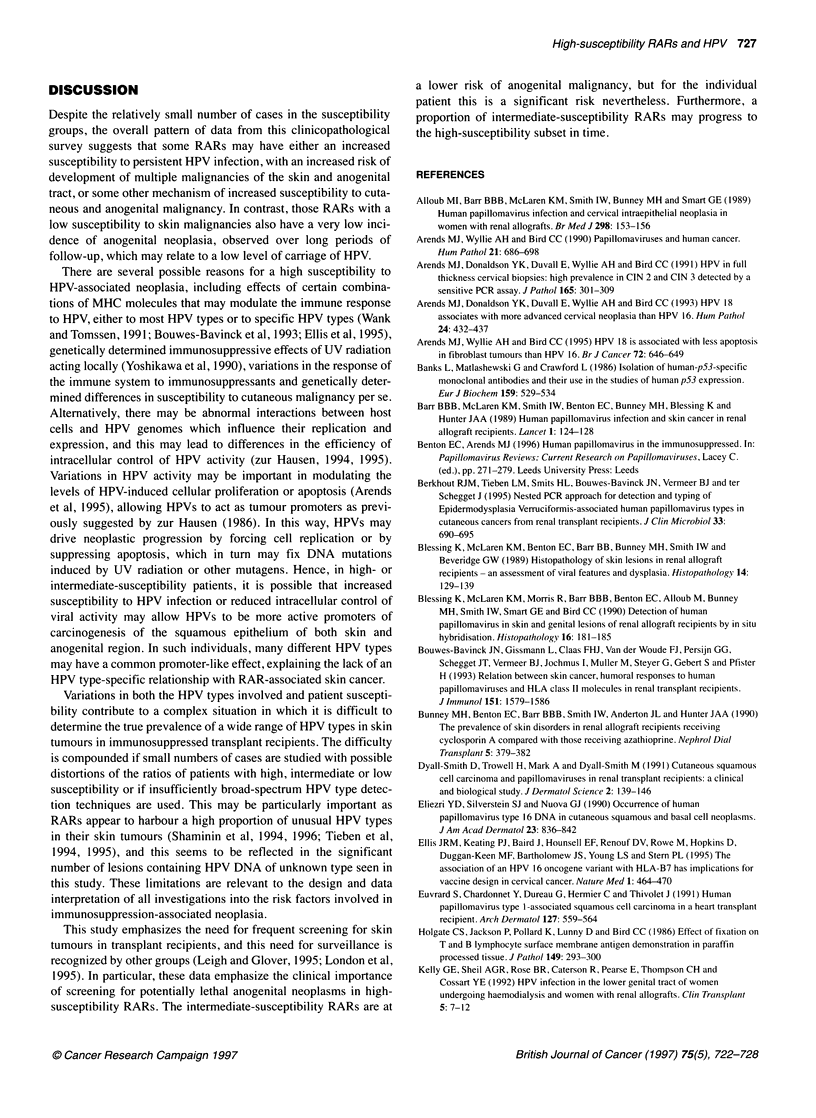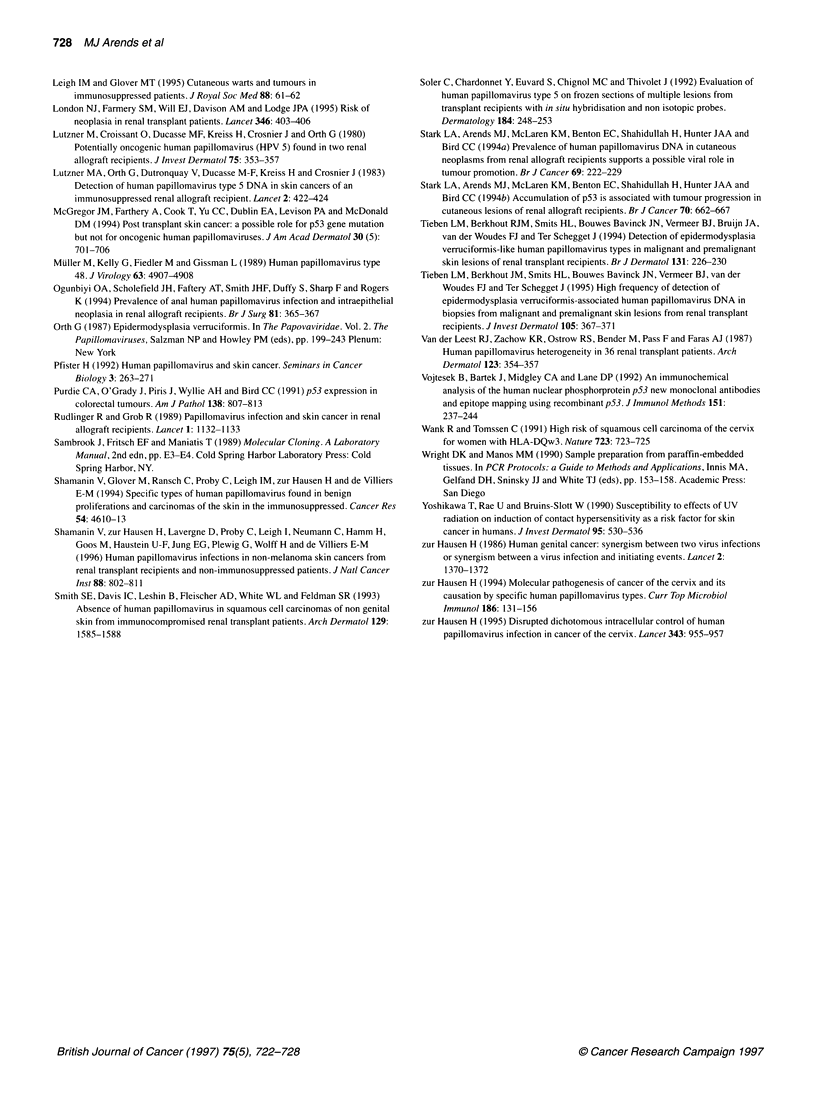# Renal allograft recipients with high susceptibility to cutaneous malignancy have an increased prevalence of human papillomavirus DNA in skin tumours and a greater risk of anogenital malignancy.

**DOI:** 10.1038/bjc.1997.128

**Published:** 1997

**Authors:** M. J. Arends, E. C. Benton, K. M. McLaren, L. A. Stark, J. A. Hunter, C. C. Bird

**Affiliations:** Department of Pathology, Edinburgh University Medical School, UK.

## Abstract

Renal allograft recipients (RARs) have a well-documented increased incidence of viral warts and cutaneous neoplasia, particularly those with long graft life and high sun exposure. A clinicopathological survey of 69 RARs in south-east Scotland, with follow-up periods of up to 28 years after transplantation, revealed marked variation in patient susceptibility to cutaneous malignancy with concomitant variation in HPV prevalence. Skin cancers were found in 34 patients. Eight patients showed high susceptibility [defined as more than four intraepidermal carcinomas (IECs) or invasive squamous cell carcinomas (SCCs)] 42 had intermediate susceptibility (1-3 IECs or SCCs, or >3 keratoses) and 18 had low susceptibility (< or = 3 keratoses and no cancers). SCCs, IECs and keratoses from the high-susceptibility group were found to have greater prevalences of human papillomavirus (HPV) DNA (56%, 45% and 50% respectively), than SCCs (0%) and IECs (33%) from intermediate-susceptibility RARs and keratoses (36%) from the combined intermediate- and low-susceptibility groups and compared with a group of immunocompetent controls (27%, 20% and 15% respectively). No differences in p53 protein accumulation, determined immunohistochemically, were observed in tumours from the three groups. Categorization of RARs by susceptibility to cutaneous malignancy provides clinically useful information, as significantly more high-susceptibility patients (38%) developed aggressive, potentially lethal anogenital or cutaneous squamous cell cancers than did patients in the intermediate group (5%, P=0.005) or the low-susceptibility group (0%).


British Journal of Cancer (1997) 75(5), 722–728
© 1997 Cancer Research Campaign

### Renal allograft recipients with high susceptibility to cutaneous malignancy have an increased prevalence of human papillomavirus DNA in skin tumours and a greater risk of anogenital malignancy

**MJ Arends[1], EC Benton[2], KM McLaren[1], LA Stark, JAA Hunter[2] and CC Bird[1]**

[1]Department of Pathology, Edinburgh University Medical School, Teviot Place, Edinburgh, UK; [2]Department of Dermatology, Royal Infirmary of Edinburgh, Lauriston Place, Edinburgh, UK

**Summary** Renal allograft recipients (RARs) have a well-documented increased incidence of viral warts and cutaneous neoplasia, particularly those with long graft life and high sun exposure. A clinicopathological survey of 69 RARs in south-east Scotland, with follow-up periods of up to 28 years after transplantation, revealed marked variation in patient susceptibility to cutaneous malignancy with concomitant variation in HPV prevalence. Skin cancers were found in 34 patients. Eight patients showed high susceptibility [defined as more than four intraepidermal carcinomas (IECs) or invasive squamous cell carcinomas (SCCs)] 42 had intermediate susceptibility (1–3 IECs or SCCs, or >3 keratoses) and 18 had low susceptibility (≤3 keratoses and no cancers). SCCs, IECs and keratoses from the high-susceptibility group were found to have greater prevalences of human papillomavirus (HPV) DNA (56%, 45% and 50% respectively), than SCCs (0%) and IECs (33%) from intermediate-susceptibility RARs and keratoses (36%) from the combined intermediate- and low-susceptibility groups and compared with a group of immunocompetent controls (27%, 20% and 15% respectively). No differences in p53 protein accumulation, determined immuno-histochemically, were observed in tumours from the three groups. Categorization of RARs by susceptibility to cutaneous malignancy provides clinically useful information, as significantly more high-susceptibility patients (38%) developed aggressive, potentially lethal anogenital or cutaneous squamous cell cancers than did patients in the intermediate group (5%, $P$=0.005) or the low-susceptibility group (0%).

**Keywords:** renal allograft recipient; human papillomavirus; keratoses; intraepidermal carcinoma; squamous cell carcinoma; viral wart; skin neoplasia; Southern hybridization; polymerase chain reaction; p53; immunocytochemistry

Renal allograft recipients (RARs) receive immunosuppressive therapy over long periods of time and have an increased incidence of cutaneous neoplasia, particularly those with a long graft life or high sun exposure (Arends et al, 1990; Benton and Arends, 1996). Allograft recipients (mainly women) are especially susceptible to human papillomavirus (HPV)-related anogenital tract neoplasia, which may be life-threatening (Alloub et al, 1989). The skin neoplasms form part of a spectrum, encompassing viral warts showing dysplasia, actinic or verrucous keratoses displaying various viral architectural features or epidermal dysplasia and sometimes in topographical continuity with intraepidermal carcinoma (IEC) and invasive squamous cell carcinoma (SCC) (Blessing et al, 1989). In contrast to anogenital cancers, 70–80% of which contain HPV 16 or 18 DNA, and to the skin cancers seen in epidermodysplasia verruciformis (EV), 90% of which contain HPV 5 or 8 DNA (Orth, 1987; Arends et al, 1990, 1993; Pfister, 1992), the results of HPV DNA detection studies in cutaneous SCCs from RARs show little consistency either in the frequency or viral type of HPV DNA found (Benton and Arends, 1996). Some of this variation may be explained by small size of study sample or differences in substrates and techniques used, i.e. fresh or frozen tissue *vs* formalin-fixed paraffin-processed tissue (associated with poorer quality of extracted DNA, particularly for amplification of longer DNA fragments) (Wright and Manos, 1990), dot blotting *vs* Southern hybridization or PCR techniques and, finally, the different ranges of HPV probes or PCR primers used.

*Received 8 March 1996*
*Revised 20 August 1996*
*Accepted 16 September 1996*

*Correspondence to*: MJ Arends, Department of Pathology, Edinburgh University Medical School, Teviot Place, Edinburgh EH8 9AG, UK

Early studies were small and focused on HPV types 5 and 8 to determine whether skin cancers of RARs resembled those of EV patients. These studies reported the detection of HPV 5 and 8 in small numbers of cases that often included highly selected individuals, such as patients with multiple SCCs (Lutzner et al, 1980, 1983; Van der Leest et al, 1987; Barr et al, 1989; Blessing et al, 1990). Negative results were reported in a number of studies; many are explicable by the use of less than ideal substrates, such as DNA from formalin-fixed tissue or suboptimal techniques, such as reverse blotting or dot-blot hybridization (Rudlinger and Grob, 1989; Blessing et al, 1990; Dyall-Smith et al, 1991; Smith et al, 1993; McGregor et al, 1994), or the use of PCR with L1 consensus primers that were designed primarily for detection of anogenital rather than cutaneous HPV types. These factors may account for negative results when looking for low copy numbers of cutaneous and EV types of HPV DNA using poor-quality substrate.

Recently, more sensitive techniques have detected a wide variety of HPV types, including uncharacterized HPV types, in skin cancers from RARs. In situ hybridization, Southern hybridization and PCR techniques detected HPV DNA in 37–54% SCCs from RARs, including anogenital, cutaneous and EV types of HPV (Müller et al, 1989; Eliezri et al, 1990; Euvrard et al, 1991;

**Table 1** Clinical and pathological data

| Patient details | | | | | Cutaneous lesions | | | | | | | |
|---|---|---|---|---|---|---|---|---|---|---|---|---|
| Code | Age/sex (M/F) | Graft (years) | Sun | Aza/ CyA | CLIN VW | Ker. | BC | IEC | SCC | IEC + SCC | Susc. | Anogenital or aggressive les. |
| *Patients with a graft life of > 15 years* | | | | | | | | | | | | |
| 16 | 55 M | 19 | Mod | Aza | W | Km | – | 9 | 14 | 23 | High | |
| 20 | 40 F | 22 | Low | Aza | W | KmE | – | 4 | 12 | 16 | High | ECis + SCC-A |
| 23 | 61 F | 25 | Mod | Aza | W | Km | 1 | 10 | 2 | 12 | High | ECis + SCC-V |
| 34 | 49 M | 28 | High | Aza | W | Km | – | 10 | 27 | 37 | High | Aggr. SCC-S |
| 54 | 57 M | 17 | High | Aza | W | Km | 1 | 2 | 8 | 10 | High | |
| 82 | 39 F | 16 | Low | Aza | W | Km | – | – | – | 0 | Int. | CIN3 + SCC-VA |
| 3 | 43 M | 20 | Low | Aza | W | Km | – | – | – | 0 | Int. | |
| 12 | 60 F | 17 | Low | Aza | W | Km | – | – | 1 | 1 | Int. | |
| 21 | 63 M | 25 | High | Aza | W | Km | – | – | – | 0 | Int. | |
| 30 | 57 F | 16 | Low | Aza | W | Km | – | – | – | 0 | Int. | |
| 46 | 45 M | 18 | High | Aza | W | Km | – | – | – | 0 | Int. | |
| 49 | 50 F | 28 | Low | Aza | W | Km | 1 | 3 | – | 3 | Int. | |
| 51 | 40 M | 21 | Low | Az + C | W | Km | – | – | – | 0 | Int. | |
| 57 | 52 M | 17 | Mod | Aza | W | Km | – | – | – | 0 | Int. | |
| 60 | 44 F | 17 | Low | Aza | W | Km | – | – | – | 0 | Int. | |
| 61 | 59 F | 16 | Low | CyA | W | K | – | – | 2 | 2 | Int. | |
| 80 | 62 M | 19 | Low | Aza | W | K | – | – | 1 | 1 | Int. | |
| 1 | 47 M | 17 | Mod | Aza | W | K | – | – | – | 0 | Low | |
| 7 | 34 F | 16 | Low | Aza | W | – | – | – | – | 0 | Low | |
| 22 | 38 F | 16 | Low | Aza | W | K | – | – | – | 0 | Low | CIN1 |
| 41 | 47 M | 23 | Low | Aza | W | K | – | – | – | 0 | Low | |
| 70 | 52 M | 17 | Mod | Aza | W | K | – | – | – | 0 | Low | |
| 71 | 43 M | 16 | Mod | Aza | W | – | – | – | – | 0 | Low | |
| 72 | 58 M | 16 | High | Aza | W | – | – | – | – | 0 | Low | |
| 73 | 38 M | 17 | Mod | Aza | W | – | – | – | – | 0 | Low | |
| 74 | 43 M | 17 | Low | Aza | W | – | – | – | – | 0 | Low | |
| 75 | 42 M | 20 | Low | Aza | W | – | – | – | – | 0 | Low | |
| 76 | 41 M | 22 | Low | Aza | – | – | – | – | – | 0 | Low | |
| 77 | 60 M | 21 | Low | Aza | W | K | – | – | – | 0 | Low | |
| 78 | 35 F | 21 | Low | Aza | W | – | – | – | – | 0 | Low | |
| 83 | 43 F | 28 | Low | Az + C | W | K | 1 | – | – | 0 | Low | |
| *Patients exhibiting keratoses, intraepidermal carcinomas or squamous cell carcinomas with a graft life of ≤ 15 years* | | | | | | | | | | | | |
| 26 | 54 M | 10 | High | Aza | W | Km | – | 6 | 15 | 21 | High | |
| 27 | 67 M | 8 | High | CyA | – | Km | – | 3 | 2 | 5 | High | |
| 64 | 57 M | 9 | High | Aza | W | Km | 1 | 4 | 3 | 7 | High | |
| 2 | 67 F | 15 | Low | Aza | W | Km | – | – | – | 0 | Int. | ECis |
| 4 | 68 M | 11 | Low | Aza | W | Km | – | – | 1 | 1 | Int. | |
| 5 | 43 F | 10 | Low | Aza | W | Km | – | 1 | – | 1 | Int. | CIN2 |
| 11 | 35 F | 11 | Low | Aza | W | K | – | 1 | – | 1 | Int. | CIN2 + VIN1 |
| 6 | 59 M | 4 | Mod | CyA | W | Km | – | – | 2 | 2 | Int. | |
| 9 | 47 M | 14 | Mod | Aza | W | K | – | – | – | 0 | Int. | |
| 56 | 63 M | 11 | Mod | Aza | W | K | 1Pr | – | – | 0 | Int. | |
| 42 | 50 M | 14 | High | Aza | W | – | – | 1 | – | 1 | Int. | |
| 84 | 69 M | 5 | High | Aza | – | K | – | 1 | – | 1 | Int. | |
| 85 | 68 F | 1 | UK | CyA | – | K | – | 1 | – | 1 | Int. | |
| 86 | 58 M | 5 | Mod | CyA | W | – | – | 1 | – | 1 | Int. | |
| 87 | 65 M | 7 | Mod | CyA | – | K | – | – | 1 | 1 | Int. | |
| 88 | 68 M | 9 | High | CyA | W | K | – | – | 1 | 1 | Int. | |
| 8 | 59 M | 8 | Mod | CyA | – | Km | – | 2 | – | 2 | Int. | |
| 13 | 59 M | 7 | High | CyA | W | K | – | 1 | – | 1 | Int. | |
| 14 | 60 M | 6 | Low | CyA | W | – | – | 1 | – | 1 | Int. | |
| 18 | 59 M | 10 C | Mod | C + Az | W | Km | – | – | – | 0 | Int. | |
| 19 | 59 F | 3 | M/Hi | CyA | – | Km | 1Pr | – | – | 0 | Int. | CIN1 |
| 35 | 45 F | 14 | Low | Aza | W | Km | – | – | – | 0 | Int. | |
| 39 | 59 M | 9 C | UK | CyA | – | Km | – | – | 2 | 2 | Int. | |
| 40 | 69 M | 15 | High | Aza | W | Km | – | 1 | – | 1 | Int. | |
| 44 | 49 F | 15 | Low | Aza | W | KmE | – | – | – | 0 | Int. | |
| 48 | 51 M | 6 | High | CyA | W | – | 5 | – | – | 0 | Int.(S) | |
| 50 | 66 M | 6 | High | Aza | – | Km | – | – | 1 | 1 | Int. | |
| 52 | 61 M | 11 | High | Aza | – | KmI | – | – | – | 1 | Int. | |
| 53 | 38 M | 8 | High | CyA | W | – | – | 1 | – | 1 | Int. | |
| 55 | 67 M | 13 | High | Aza | W | Km | – | – | 1 | 1 | Int. | |
| 58 | 78 M | 11 | High | Aza | – | Km | – | 2 | – | 2 | Int. | |
| 59 | 71 M | 3 | Mod | CyA | – | – | 3 | 1 | – | 1 | Int. | |
| 62 | 63 M | 15 | Mod | Aza | W | Km | 1 | 1 | 2 | 3 | Int. | |
| 31 | 30 F | 13 | Mod | Aza | W | K | – | – | – | 0 | Low | |
| 33 | 31 F | 11 | Low | Aza | – | K | – | – | – | 0 | Low | CIN1 |
| 10 | 31 M | 10 | Low | Az + C | W | K | – | – | – | 0 | Low | |
| 17 | 44 M | 14 | Mod | Aza | W | – | – | – | – | 0 | Low | |
| 81 | 54 M | 12 | High | Aza | W | K | – | – | – | 0 | Low | |

Accumulated lesions of transplant recipients up to the end of 1994. Sex: M/F = Male/Female. Graft C, cardiac allograft recipient; all others are renal allograft recipients. Sun exposure ratings: low, very little; mod, outdoor leisure activities; high, outdoor occupation of more than 3 months or lived in a tropical climate; M/Hi, mod to high rating; UK, unknown. Immunosuppressants: Aza, azathioprine; CyA, cyclosporin A; C + Az, both CyA and Aza. CLIN, clinical observations (over long term follow-up) of cutaneous lesions (not always biopsied); VW, viral warts; W, multiple warts; Ker., keratoses; K, 1–3 keratoses; Km, multiple keratoses (more than 3); E, epidermodysplasia verruciformis-like plaque; I, intraepidermal carcinoma diagnosed clinically without histology. Histological diagnosis: BC, basal cell carcinoma; IEC, intraepidermal carcinoma; SCC, squamous cell carcinoma; IEC + SCC, combined total of cutaneous malignancies including intraepidermal carcinomas and squamous cell carcinomas. Susc., susceptibility to development of cutaneous malignancy; high susceptibility defined histopathologially as ≥ 4 IEC/SCC; Int., intermediate susceptibility defined as 1–3 IEC/SCC or >3 keratoses (including moderate or severe dysplasia within keratoses); Low, low susceptibility defined as non-dysplastic warts or 1–3 keratoses (showing mild to moderate dysplasia), including clinically observed, non-biopsied keratoses. Anogenital or Aggressive les., anogenital or aggressive cutaneous lesions; patient 20 had extensive carcinoma in situ (ECis) with CIN3 of cervix, VIN3 of vulva, AIN3 of anal canal and also developed an invasive squamous cell carcinoma of anal canal [SCC-A] that metastasized; patient 23 had extensive carcinoma in situ (ECis) with CIN3 of cervix and VIN3 of vulva that developed into an invasive squamous cell carcinoma of vulva [SCC-V]; patient 34 developed multiple and confluent squamous cell carcinomas of the scalp with aggressive invasion of scalp muscle and skull (Aggr. SCC-S); patient 82 developed CIN3 and squamous cell carcinoma of the vulva and anus/perineum (CIN3 + SCC-VA); patient 22 developed CIN 1 of the cervix. Patient 2 had extensive carcinoma in situ (ECis) with CIN3 of cervix, VAIN3 of vagina and VIN3 of vulva. Patient 48 appears to have an intermediate susceptibility of special type [Int. (S)], developing five BCCs but no IECs or SCCs; patient 5 developed CIN 2 of the cervix; patient 11 developed CIN2 of cervix and VIN1 of vulva; and patients 19 and 33 developed CIN 1. 1 Pr, 1 prior lesion occurring before transplantation.

© Cancer Research Campaign 1997 

Soler et al, 1992; Stark et al, 1994*a*). Uncharacterized HPV types have been found in benign and malignant skin tumours using either degenerate PCR primers (Shamanin et al, 1994), multiple complementary sets of consensus PCR primers (Tieben et al, 1994) or nested PCR assays (Berkhout et al, 1995). The nested PCR approach detected HPV in 81% SCCs from RARs, including a wide spectrum of known and novel EV types of HPV (Berkhout et al, 1995). It appears that, as HPV diagnostic technology for a broad spectrum of HPV types has improved, the reported prevalence of HPV DNA in SCCs from RARs has crept upwards and currently stands at around 80%. Here, we present data from a clinicopathological survey of RARs in south-east Scotland. This showed a widely disparate susceptibility to cutaneous malignancy among RARs, with evidence of concomitant variation in HPV DNA prevalence in their skin neoplasms. This suggests an important source of bias that may affect small studies and provides an explanation for the reported variation in HPV DNA prevalence and the wide range of viral types in other studies. Significantly, high susceptibility for cutaneous malignancy may represent an early clinical marker for increased risk of development of potentially lethal anogenital or aggressive cutaneous malignancy.

### METHODS

### Patients

Sixty-nine RARs and 53 immunocompetent patients (ICPs) were investigated. RARs received transplants between 1965 and 1994. Before 1984, prednisolone and azathioprine were the main immunosuppressive drugs used, but thereafter most patients received prednisolone and cyclosporin A. Immunocompetent patients all presented to the Dermatology Department in Edinburgh Royal Infirmary, UK, for treatment of viral warts or skin tumours. Most of these patients were elderly with lesions on sun-exposed sites.

### Tissue collection

Biopsy samples were bisected longitudinally, half was placed immediately in PLPD (periodate lysine paraformaldehyde dichromate) (Holgate et al, 1986) or 10% formalin and fixed for 24 h at 4°C before paraffin embedding. Histological assessment and immunohistochemistry were carried out on sections prepared from paraffin-embedded material. The other half were snap frozen in liquid nitrogen and stored at – 70°C to await DNA extraction and virological investigation.

### DNA extraction and HPV detection

Frozen tissue was minced in lysis buffer (50 mM Tris, 50 mM EDTA, 100 mM sodium chloride, 5 mM DTT, 1% sodium dodecyl sulphate (SDS), 1.5 mg $ml^{-1}$ proteinase K) then incubated at 37°C overnight; DNA extraction was carried out using a standard phenol–chloroform extraction technique (Sambrook et al, 1989). Two methods were used to screen for the presence of HPV DNA (Stark et al, 1994*a*). Low-stringency Southern hybridization analysis, using mixed HPV probes at low hybridization ($T_m$ – 40°C) and washing stringency ($T_m$ –35°C), was used to detect a range of common cutaneous and epidermodysplasia verruciformis (EV)-related types, including HPV types 1–20. Highly sensitive polymerase chain reaction (PCR) assays were used to detect specific HPV types 1, 2, 5, 8, 6b, 11, 16 and 18 (Arends et al, 1991; Stark et al, 1994*a*).

### Histopathology

The skin lesions were classified as follows: viral warts (VWs) exhibited symmetry, papilliferous architecture and koilocytic change; verrucous keratoses (VKs) displayed the architecture of warts but lacked definitive cytological features of viral infection; and actinic keratoses (AKs) showed basal budding and basal atypia (degrees of dysplasia were assessed in both types of keratosis); however, actinic keratoses and verrucous keratoses were combined into a single group of keratoses (Ker.) for analysis of data in this study. Intraepidermal carcinoma (IEC) showed either full-thickness dysplasia or severe dysplasia and acantholysis of the basal layer; invasive squamous cell carcinoma (SCC) showed definite dermal invasion (Blessing et al, 1989).

### Immunocytochemical and mutational analysis of p53

Immunocytochemistry was performed on 3-μm sections of PLPD- and formalin-fixed tissue, using the mouse anti p53 monoclonal antibodies MAb Do-7 (Vojtesek et al, 1992) and PAb 1801 (Banks et al, 1986) and a standard ABC horseradish peroxidase (HRP) technique (Dako, High Wycombe, Bucks, UK) as previously described (Purdie et al, 1991; Stark et al, 1994*b*). Formalin-fixed tissue was treated with MAb Do-7 (1:100 dilution, overnight incubation) only, whereas PLPD-fixed material was treated with MAb Do-7 and PAb 1801 (1:100 dilution, 1-h incubation). Single-strand conformational polymorphism (SSCP) analysis for mutations in exons 5–8 of the *p53* gene was performed on selected cases as previously described (Stark et al, 1994*b*).

### RESULTS

### Clinicopathological survey of cutaneous lesions in renal allograft recipients

We report a survey of RARs from the south-east of Scotland, who have been monitored dermatologically over the past 15 years to facilitate early detection of infective, premalignant or malignant cutaneous lesions. Records have been kept of all skin lesions developing since transplantation, and suspicious lesions have been biopsied. Data have been collated (Table 1) for a group of 69 transplant patients, of whom 34 developed cutaneous malignancies. Eight RARs were highly susceptible to the development of malignant squamous tumours of the skin – defined clinicopathologically as four or more IECs or SCCs. These high-susceptibility RARs developed 5–37 cutaneous malignancies (IECs and SCCs) each, with a median of 14 per patient. Forty-two RARs showed an intermediate susceptibility to cutaneous malignancy, defined as 1–3 IECs or SCCs or >3 keratoses, both actinic and verrucous, including those with moderate or severe epidermal dysplasia. Intermediate susceptibility RARs developed a median of one cutaneous malignancy each. Nineteen RARs showed a low susceptibility to cutaneous malignancy defined as no IECs, no SCCs and ≤3 keratoses (showing only mild or moderate dysplasia if biopsied) over a period of at least 10 years since transplantation, despite developing multiple warts in some cases (Table 1).

Thirty-one RARs had graft lives of greater than 15 years. A comparison of skin tumours from these patients with those from

 © Cancer Research Campaign 1997

**Table 2** HPV-positive lesions from RAR

| Patient code | Susceptibility | Histology | Southern hybridization | PCR |
|---|---|---|---|---|
| 16 | High | SCC | Pos(2) | 2 |
| | | SCC | Pos(UK) | Neg |
| | | IEC | Neg | 16 |
| 20 | High | SCC | Neg | 6 |
| | | SCC | Neg | 16 |
| | | SCC | Neg | 2 |
| | | SCC | Pos(UK) | Neg |
| | | IEC | Pos(UK) | Neg |
| | | IEC | Pos(5) | 5 |
| | | IEC | Pos(3) | Neg |
| | | Ker. | ND | 5 |
| 23 | High | SCC | Pos(UK) | Neg |
| | | IEC | Pos(UK) | Neg |
| | | IEC | Neg | 16 |
| 34 | High | SCC | Pos(UK) | Neg |
| | | SCC | Pos(UK) | Neg |
| | | SCC | Pos(UK) | Neg |
| | | IEC | Neg | 2 |
| | | IEC | Pos(UK) | Neg |
| 54 | High | Ker. | Neg | 5 |
| | | Ker. | Neg | 1 |
| | | Ker. | Neg | 1 |
| | | Ker. | Pos(UK) | Neg |
| | | Ker. | Neg | 2 |
| 26 | High | SCC | Pos(3) | Neg |
| | | SCC | Pos(UK) | Neg |
| | | SCC | Pos(UK) | Neg |
| | | SCC | Pos(1) | 1 |
| | | IEC | Pos(UK) | Neg |
| | | Ker. | Pos(UK) | Neg |
| 64 | High | SCC | Pos(UK) | Neg |
| | | IEC | Pos(UK) | Neg |
| | | Ker. | Pos(UK) | Neg |
| 8 | Int. | IEC | Pos(UK) | Neg |
| | | Ker. | Pos(UK) | Neg |
| 18 | Int. | Ker. | Pos(UK) | Neg |
| | | Ker. | Pos(10) | Neg |
| 40 | Int. | IEC | Neg | 2 |
| 50 | Int. | Ker. | Neg | 16 |
| | | Ker. | Pos(UK) | Neg |
| 59 | Int. | IEC | Pos(UK) | Neg |

Int., intermediate; SCC, squamous cell carcinoma; IEC, intraepidermal carcinoma; Ker., keratosis; pos(2), positive (HPV type 2); pos(UK), positive (unknown HPV type); neg, negative.

38 RARs with a graft life of ≤15 years revealed no apparent differences in the prevalences of HPV DNA (Table 2). The group of patients with graft lives of >15 years included five high-susceptibility patients. The remaining three high-susceptibility patients had graft lives of 8–10 years. The median graft lives for the three patient groups were similar, as were their median ages (Tables 1 and 3). Despite the small numbers of patients concerned, these data suggest that long graft life is not the only determinant of the high-susceptibility phenotype, although the passage of time appears to be required for its expression. Thus, it is possible that some of the currently regarded 'intermediate-susceptibility' patients with short graft lives may eventually go on to develop large numbers of IECs or SCCs when sufficient time has elapsed to allow expression of the high-susceptibility phenotype. However, 14 out of 19 low-susceptibility patients had grafts of > 15 years' duration and thus appear to have a low risk of cutaneous malignancy that is stable.

**Figure 1** HPV DNA prevalence in seven renal allograft recipients with high susceptibility to cutaneous malignancies (■, RAR-HS) from which lesional DNA was available for HPV DNA detection using both Southern hybridization analysis and PCR. These are compared with 23 intermediate- or low-susceptibility renal allograft recipients (▨, RAR-ILS) and 53 immunocompetent patient controls (□, ICP) in three groups of cutaneous neoplasms collected between 1989 and 1993, including keratoses (Ker.), intraepidermal carcinomas (IEC) and squamous cell carcinomas (SCC)

Seven high-susceptibility patients received immunosuppressive therapy based primarily on azathioprine, and one received cyclosporin A, but this reflected the availability of immunosuppressive drugs at the time of transplantation (cyclosporin A was introduced in 1984/85). Insufficient time has elapsed for valid comparisons to be made between patients receiving azathioprine and those treated with cyclosporin A, although no significant differences were found between the two regimens at 4 years (Bunney et al, 1990). Furthermore, drug doses have been modified over this extended study period, as has advice given to patients regarding protection against sun exposure. The ranges of patients' ages and sun-exposure ratings between the different susceptibility groups overlap to such an extent that, given the small numbers of patients involved, no definite pattern of variation emerges.

### HPV prevalence and patient susceptibility

To investigate HPV DNA prevalence, 159 cutaneous biopsies were collected during the period 1989–93 from a sample of the survey group that included 19 women and 33 men. Histopathological analysis showed 28 viral warts, 48 keratoses (combined actinic and verrucous), 35 IECs, 41 SCCs and seven basal cell carcinomas, diagnosed by previously described criteria (Blessing et al, 1989). The IECs and SCCs examined were collected from 18 RARs. Control samples were collected over the same period and included 103 biopsies from immunocompetent patients (ICPs), including 48 with either IEC or SCC; these patients were, on average, 20 years older (mean age 75 years) than RARs with similar lesions (mean age 54 years). All of these lesions were subjected to HPV DNA detection by both low-stringency Southern hybridization analysis, capable of detecting almost all of HPV types 1–20, and PCR assays specific for HPV types 1, 2, 5, 8, 6b, 11, 16 and 18, as previously described (Arends et al, 1991; Stark et al, 1994*a*). These analyses showed a range of different HPV types within the spectrum of skin neoplasms, including several of unknown HPV type detected by low-stringency Southern hybridization analysis, but no specific pattern or combination of HPV types in any one lesion (Table 2).

© Cancer Research Campaign 1997

**Table 3** Characteristics of the 3 susceptibility groups

| Susceptibility | No. of patients age range, median (years) | Graft life range median (years) | Malignant histology IEC + SCC: Range | Malignant histology IEC + SCC: Total (median no. of lesions per case) | No. of patients (%) with anogenital or aggressive skin lesions |
|---|---|---|---|---|---|
| High | 8<br>26–61, 54 | 8–28, 18 | 5–37 | 131 (14) | 3 (38) |
| Intermediate | 42<br>35–78, 59 | 1–28, 11 | 0–3 | 35 (1) | 2 (5) |
| Low | 19<br>30–60, 43 | 10–28, 17 | 0 | 0(0) | 0 (0) |

Susceptibility, susceptibility to development of cutaneous malignancy, high susceptibility defined histopathologically as ≥ 4 IEC/SCC; intermediate susceptibility defined as 1–3 IEC/SCC or > 3 keratoses (including those with moderate or severe dysplasia within keratoses); low, low susceptibility defined as non-dysplastic warts or 1–3 keratoses (showing mild/moderate dysplasia), including clinically observed, non-biopsied keratoses with no IECs and no SCCs over a period of at least 10 years after transplantation. Malignant histology (lesions up to the end of 1994 included): IEC + SCC, combined total of cutaneous malignancies including intraepidermal carcinomas (IEC) and squamous cell carcinomas (SCC). Anogenital or aggressive skin lesions, see text for details.

The overall prevalences of HPV DNA are shown in Figure 1 for seven high-susceptibility patients, 23 intermediate- or low-susceptibility patients (these two groups were combined because of small specimen numbers of keratoses for analysis) and 53 immunocompetent patient controls. HPV DNA prevalence was 50% in keratoses (8/16), 45% in IECs (10/22) and 56% in SCCs (15/27) from the high-susceptibility RARs, and these were greater than those from either intermediate- or low-susceptibility RARs – 36% in keratoses (5/14 from both intermediate- and low-susceptibility RARs), 33% in IECs (3/9 from intermediate-susceptibility RARs) and 0% in SCCs (0/2 from intermediate-susceptibility RARs) and were also greater than those seen in immunocompetent patients: 15% in keratoses (2/13), 20% in IECs (5/25) and 27% in SCCs (4/15). Statistical comparisons using the chi-squared test showed significant differences only for HPV DNA prevalence in keratoses from the high-susceptibility subset compared with that in immunocompetent patients' keratoses ($P<0.05$), although an interesting, but not statistically significant, trend was seen in the comparison of the differences between HPV prevalences in the three groups of SCCs (high-susceptibility subset, intermediate-susceptibility subset and immunocompetent patients) ($P=0.0875$); the small numbers of cases limited the power of this analysis. There may be some under-reporting of HPV prevalence using this combination of low-stringency Southern hybridization analysis and specific PCR assays as only 9/14 viral warts from RARs were shown to contain HPV DNA using these methods, although this did increase to 14/19 (74%) if viral warts from immunocompetent patients were included.

### Accumulation of p53 protein in skin neoplasms

Accumulation of the p53 protein, a tumour suppressor involved in the response to DNA damage, was analysed in a large subset of these specimens from different susceptibility groups. One hundred and twenty-eight biopsies of skin tumours from RARs and 75 from ICPs were screened for both p53 immunoreactivity and the presence of HPV DNA (Stark et al, 1994*b*). Because of the limitations of p53 immunostaining, single-strand conformational polymorphism (SSCP) analysis of exons 5 to 8 of the *p53* gene was used in 28 malignancies (IECs and SCCs) and detected *p53* mutations in 5/9 (56%) lesions with widespread p53 immunostaining (>50% of tumour cells), 1/6 (17%) lesions with p53 staining occurring in 10–50% of cells and none when <10% cells were stained (Stark et al, 1994*b*). No clear relationship was observed between the presence or extent of accumulated p53 protein or *p53* mutations and either presence of HPV DNA or patient susceptibility. Thus, these data excluded p53 accumulation as a useful pathological marker of the high-susceptibility phenotype.

### Anogenital neoplasia and patient susceptibility

A statistically significant difference was observed in the occurrence of potentially life-threatening multiple anogenital tract neoplasms or aggressive cutaneous malignancy in 38% (3/8) high-susceptibility RARs compared with 5% (2/42) intermediate-susceptibility RARs ($P=0.005$, chi-squared test). There were only two women among the eight high-susceptibility RARs and both developed extensive anogenital carcinoma in situ (two or more of CIN 3 of cervix, VAIN 3 of vagina, VIN 3 of vulva and AIN 3 of anal canal), together with an invasive squamous cell carcinoma of either vulva, perineum or anal canal (one patient died of metastatic anogenital carcinoma and one is still alive), whereas only 2 of 13 (15%) women in the intermediate-susceptibility RARs developed in situ carcinoma or invasive SCCs of the anogenital region (one patient died of metastatic anogenital carcinoma and one died of a cerebrovascular accident) (Table 1). A male high-susceptibility RAR developed lethal, highly aggressive, spindle cell cutaneous SCCs (multiple and confluent SCCs of scalp with aggressive local invasion into scalp muscle, skull and dura mater). Two intermediate-susceptibility RARs developed CIN2, one with VIN1 also. No high-grade anogenital lesions were found in the low-susceptibility patients. Two low- and one intermediate-susceptibility patients developed CIN1. HPV DNA prevalence was not assessed in these lesions because fresh samples were not available. These data are consistent with previous findings of a higher prevalence of CIN in female RARs than in ICP controls, with increased detection of 'high risk' HPV 16 and 18 in CIN lesions and subclinical HPV infections from RARs than controls (Alloub et al, 1989; Kelly et al, 1991) and also with a higher risk of anal HPV infection and anal neoplasia in RARs than controls (Ogunbiyi et al, 1994).

© Cancer Research Campaign 1997

### DISCUSSION

Despite the relatively small number of cases in the susceptibility groups, the overall pattern of data from this clinicopathological survey suggests that some RARs may have either an increased susceptibility to persistent HPV infection, with an increased risk of development of multiple malignancies of the skin and anogenital tract, or some other mechanism of increased susceptibility to cutaneous and anogenital malignancy. In contrast, those RARs with a low susceptibility to skin malignancies also have a very low incidence of anogenital neoplasia, observed over long periods of follow-up, which may relate to a low level of carriage of HPV.

There are several possible reasons for a high susceptibility to HPV-associated neoplasia, including effects of certain combinations of MHC molecules that may modulate the immune response to HPV, either to most HPV types or to specific HPV types (Wank and Tomssen, 1991; Bouwes-Bavinck et al, 1993; Ellis et al, 1995), genetically determined immunosuppressive effects of UV radiation acting locally (Yoshikawa et al, 1990), variations in the response of the immune system to immunosuppressants and genetically determined differences in susceptibility to cutaneous malignancy per se. Alternatively, there may be abnormal interactions between host cells and HPV genomes which influence their replication and expression, and this may lead to differences in the efficiency of intracellular control of HPV activity (zur Hausen, 1994, 1995). Variations in HPV activity may be important in modulating the levels of HPV-induced cellular proliferation or apoptosis (Arends et al, 1995), allowing HPVs to act as tumour promoters as previously suggested by zur Hausen (1986). In this way, HPVs may drive neoplastic progression by forcing cell replication or by suppressing apoptosis, which in turn may fix DNA mutations induced by UV radiation or other mutagens. Hence, in high- or intermediate-susceptibility patients, it is possible that increased susceptibility to HPV infection or reduced intracellular control of viral activity may allow HPVs to be more active promoters of carcinogenesis of the squamous epithelium of both skin and anogenital region. In such individuals, many different HPV types may have a common promoter-like effect, explaining the lack of an HPV type-specific relationship with RAR-associated skin cancer.

Variations in both the HPV types involved and patient susceptibility contribute to a complex situation in which it is difficult to determine the true prevalence of a wide range of HPV types in skin tumours in immunosuppressed transplant recipients. The difficulty is compounded if small numbers of cases are studied with possible distortions of the ratios of patients with high, intermediate or low susceptibility or if insufficiently broad-spectrum HPV type detection techniques are used. This may be particularly important as RARs appear to harbour a high proportion of unusual HPV types in their skin tumours (Shaminin et al, 1994, 1996; Tieben et al, 1994, 1995), and this seems to be reflected in the significant number of lesions containing HPV DNA of unknown type seen in this study. These limitations are relevant to the design and data interpretation of all investigations into the risk factors involved in immunosuppression-associated neoplasia.

This study emphasizes the need for frequent screening for skin tumours in transplant recipients, and this need for surveillance is recognized by other groups (Leigh and Glover, 1995; London et al, 1995). In particular, these data emphasize the clinical importance of screening for potentially lethal anogenital neoplasms in high-susceptibility RARs. The intermediate-susceptibility RARs are at a lower risk of anogenital malignancy, but for the individual patient this is a significant risk nevertheless. Furthermore, a proportion of intermediate-susceptibility RARs may progress to the high-susceptibility subset in time.

### REFERENCES

Alloub MI, Barr BBB, McLaren KM, Smith IW, Bunney MH and Smart GE (1989) Human papillomavirus infection and cervical intraepithelial neoplasia in women with renal allografts. *Br Med J* **298**: 153–156

Arends MJ, Wyllie AH and Bird CC (1990) Papillomaviruses and human cancer. *Hum Pathol* **21**: 686–698

Arends MJ, Donaldson YK, Duvall E, Wyllie AH and Bird CC (1991) HPV in full thickness cervical biopsies: high prevalence in CIN 2 and CIN 3 detected by a sensitive PCR assay. *J Pathol* **165**: 301–309

Arends MJ, Donaldson YK, Duvall E, Wyllie AH and Bird CC (1993) HPV 18 associates with more advanced cervical neoplasia than HPV 16. *Hum Pathol* **24**: 432–437

Arends MJ, Wyllie AH and Bird CC (1995) HPV 18 is associated with less apoptosis in fibroblast tumours than HPV 16. *Br J Cancer* **72**: 646–649

Banks L, Matlashewski G and Crawford L (1986) Isolation of human-*p53*-specific monoclonal antibodies and their use in the studies of human *p53* expression. *Eur J Biochem* **159**: 529–534

Barr BBB, McLaren KM, Smith IW, Benton EC, Bunney MH, Blessing K and Hunter JAA (1989) Human papillomavirus infection and skin cancer in renal allograft recipients. *Lancet* **1**: 124–128

Benton EC, Arends MJ (1996) Human papillomavirus in the immunosuppressed. In: *Papillomavirus Reviews: Current Research on Papillomaviruses*, Lacey C. (ed.), pp. 271–279. Leeds University Press: Leeds

Berkhout RJM, Tieben LM, Smits HL, Bouwes-Bavinck JN, Vermeer BJ and ter Schegget J (1995) Nested PCR approach for detection and typing of Epidermodysplasia Verruciformis-associated human papillomavirus types in cutaneous cancers from renal transplant recipients. *J Clin Microbiol* **33**: 690–695

Blessing K, McLaren KM, Benton EC, Barr BB, Bunney MH, Smith IW and Beveridge GW (1989) Histopathology of skin lesions in renal allograft recipients – an assessment of viral features and dysplasia. *Histopathology* **14**: 129–139

Blessing K, McLaren KM, Morris R, Barr BBB, Benton EC, Alloub M, Bunney MH, Smith IW, Smart GE and Bird CC (1990) Detection of human papillomavirus in skin and genital lesions of renal allograft recipients by in situ hybridisation. *Histopathology* **16**: 181–185

Bouwes-Bavinck JN, Gissmann L, Claas FHJ, Van der Woude FJ, Persijn GG, Schegget JT, Vermeer BJ, Jochmus I, Muller M, Steyer G, Gebert S and Pfister H (1993) Relation between skin cancer, humoral responses to human papillomaviruses and HLA class II molecules in renal transplant recipients. *J Immunol* **151**: 1579–1586

Bunney MH, Benton EC, Barr BBB, Smith IW, Anderton JL and Hunter JAA (1990) The prevalence of skin disorders in renal allograft recipients receiving cyclosporin A compared with those receiving azathioprine. *Nephrol Dial Transplant* **5**: 379–382

Dyall-Smith D, Trowell H, Mark A and Dyall-Smith M (1991) Cutaneous squamous cell carcinoma and papillomaviruses in renal transplant recipients: a clinical and biological study. *J Dermatol Science* **2**: 139–146

Eliezri YD, Silverstein SJ and Nuova GJ (1990) Occurrence of human papillomavirus type 16 DNA in cutaneous squamous and basal cell neoplasms. *J Am Acad Dermatol* **23**: 836–842

Ellis JRM, Keating PJ, Baird J, Hounsell EF, Renouf DV, Rowe M, Hopkins D, Duggan-Keen MF, Bartholomew JS, Young LS and Stern PL (1995) The association of an HPV 16 oncogene variant with HLA-B7 has implications for vaccine design in cervical cancer. *Nature Med* **1**: 464–470

Euvrard S, Chardonnet Y, Dureau G, Hermier C and Thivolet J (1991) Human papillomavirus type 1-associated squamous cell carcinoma in a heart transplant recipient. *Arch Dermatol* **127**: 559–564

Holgate CS, Jackson P, Pollard K, Lunny D and Bird CC (1986) Effect of fixation on T and B lymphocyte surface membrane antigen demonstration in paraffin processed tissue. *J Pathol* **149**: 293–300

Kelly GE, Sheil AGR, Rose BR, Caterson R, Pearse E, Thompson CH and Cossart YE (1992) HPV infection in the lower genital tract of women undergoing haemodialysis and women with renal allografts. *Clin Transplant* **5**: 7–12

© Cancer Research Campaign 1997

Leigh IM and Glover MT (1995) Cutaneous warts and tumours in immunosuppressed patients. *J Royal Soc Med* **88**: 61–62

London NJ, Farmery SM, Will EJ, Davison AM and Lodge JPA (1995) Risk of neoplasia in renal transplant patients. *Lancet* **346**: 403–406

Lutzner M, Croissant O, Ducasse MF, Kreiss H, Crosnier J and Orth G (1980) Potentially oncogenic human papillomavirus (HPV 5) found in two renal allograft recipients. *J Invest Dermatol* **75**: 353–357

Lutzner MA, Orth G, Dutronquay V, Ducasse M-F, Kreiss H and Crosnier J (1983) Detection of human papillomavirus type 5 DNA in skin cancers of an immunosuppressed renal allograft recipient. *Lancet* **2**: 422–424

McGregor JM, Farthery A, Cook T, Yu CC, Dublin EA, Levison PA and McDonald DM (1994) Post transplant skin cancer: a possible role for p53 gene mutation but not for oncogenic human papillomaviruses. *J Am Acad Dermatol* **30** (5): 701–706

Müller M, Kelly G, Fiedler M and Gissman L (1989) Human papillomavirus type 48. *J Virology* **63**: 4907–4908

Ogunbiyi OA, Scholefield JH, Faftery AT, Smith JHF, Duffy S, Sharp F and Rogers K (1994) Prevalence of anal human papillomavirus infection and intraepithelial neoplasia in renal allograft recipients. *Br J Surg* **81**: 365–367

Orth G (1987) Epidermodysplasia verruciformis. In *The Papovaviridae*. Vol. 2. *The Papillomaviruses,* Salzman NP and Howley PM (eds), pp. 199–243 Plenum: New York

Pfister H (1992) Human papillomavirus and skin cancer. *Seminars in Cancer Biology* **3**: 263–271

Purdie CA, O'Grady J, Piris J, Wyllie AH and Bird CC (1991) *p53* expression in colorectal tumours. *Am J Pathol* **138**: 807–813

Rudlinger R and Grob R (1989) Papillomavirus infection and skin cancer in renal allograft recipients. *Lancet* **1**: 1132–1133

Sambrook J, Fritsch EF and Maniatis T (1989) *Molecular Cloning. A Laboratory Manual*, 2nd edn, pp. E3–E4. Cold Spring Harbor Laboratory Press: Cold Spring Harbor, NY.

Shamanin V, Glover M, Ransch C, Proby C, Leigh IM, zur Hausen H and de Villiers E-M (1994) Specific types of human papillomavirus found in benign proliferations and carcinomas of the skin in the immunosuppressed. *Cancer Res* **54**: 4610–13

Shamanin V, zur Hausen H, Lavergne D, Proby C, Leigh I, Neumann C, Hamm H, Goos M, Haustein U-F, Jung EG, Plewig G, Wolff H and de Villiers E-M (1996) Human papillomavirus infections in non-melanoma skin cancers from renal transplant recipients and non-immunosuppressed patients. *J Natl Cancer Inst* **88**: 802–811

Smith SE, Davis IC, Leshin B, Fleischer AD, White WL and Feldman SR (1993) Absence of human papillomavirus in squamous cell carcinomas of non genital skin from immunocompromised renal transplant patients. *Arch Dermatol* **129**: 1585–1588

Soler C, Chardonnet Y, Euvard S, Chignol MC and Thivolet J (1992) Evaluation of human papillomavirus type 5 on frozen sections of multiple lesions from transplant recipients with *in situ* hybridisation and non isotopic probes. *Dermatology* **184**: 248–253

Stark LA, Arends MJ, McLaren KM, Benton EC, Shahidullah H, Hunter JAA and Bird CC (1994*a*) Prevalence of human papillomavirus DNA in cutaneous neoplasms from renal allograft recipients supports a possible viral role in tumour promotion. *Br J Cancer* **69**: 222–229

Stark LA, Arends MJ, McLaren KM, Benton EC, Shahidullah H, Hunter JAA and Bird CC (1994*b*) Accumulation of p53 is associated with tumour progression in cutaneous lesions of renal allograft recipients. *Br J Cancer* **70**: 662–667

Tieben LM, Berkhout RJM, Smits HL, Bouwes Bavinck JN, Vermeer BJ, Bruijn JA, van der Woudes FJ and Ter Schegget J (1994) Detection of epidermodysplasia verruciformis-like human papillomavirus types in malignant and premalignant skin lesions of renal transplant recipients. *Br J Dermatol* **131**: 226–230

Tieben LM, Berkhout JM, Smits HL, Bouwes Bavinck JN, Vermeer BJ, van der Woudes FJ and Ter Schegget J (1995) High frequency of detection of epidermodysplasia verruciformis-associated human papillomavirus DNA in biopsies from malignant and premalignant skin lesions from renal transplant recipients. *J Invest Dermatol* **105**: 367–371

Van der Leest RJ, Zachow KR, Ostrow RS, Bender M, Pass F and Faras AJ (1987) Human papillomavirus heterogeneity in 36 renal transplant patients. *Arch Dermatol* **123**: 354–357

Vojtesek B, Bartek J, Midgley CA and Lane DP (1992) An immunochemical analysis of the human nuclear phosphorprotein *p53* new monoclonal antibodies and epitope mapping using recombinant *p53*. *J Immunol Methods* **151**: 237–244

Wank R and Tomssen C (1991) High risk of squamous cell carcinoma of the cervix for women with HLA-DQw3. *Nature* **723**: 723–725

Wright DK and Manos MM (1990) Sample preparation from paraffin-embedded tissues. In *PCR Protocols: a Guide to Methods and Applications*, Innis MA, Gelfand DH, Sninsky JJ and White TJ (eds), pp. 153–158. Academic Press: San Diego

Yoshikawa T, Rae U and Bruins-Slott W (1990) Susceptibility to effects of UV radiation on induction of contact hypersensitivity as a risk factor for skin cancer in humans. *J Invest Dermatol* **95**: 530–536

zur Hausen H (1986) Human genital cancer: synergism between two virus infections or synergism between a virus infection and initiating events. *Lancet* **2**: 1370–1372

zur Hausen H (1994) Molecular pathogenesis of cancer of the cervix and its causation by specific human papillomavirus types. *Curr Top Microbiol Immunol* **186**: 131–156

zur Hausen H (1995) Disrupted dichotomous intracellular control of human papillomavirus infection in cancer of the cervix. *Lancet* **343**: 955–957

© Cancer Research Campaign 1997